# Dendrimers as Color-Stabilizers of Pyranoanthocyanins: The Dye Concentration Governs the Host–Guest Interaction Mechanisms

**DOI:** 10.1021/acsapm.0c01321

**Published:** 2021-03-03

**Authors:** Luís Cruz, Juan Correa, Nuno Mateus, Victor de Freitas, Maun H. Tawara, Eduardo Fernandez-Megia

**Affiliations:** † REQUIMTE/LAQV, Departamento de Química e Bioquímica, Faculdade de Ciências, Universidade do Porto, Rua do Campo Alegre, s/n, 4169-007 Porto, Portugal; ‡ Centro Singular de Investigación en Química Biolóxica e Materiais Moleculares (CIQUS) and Departamento de Química Orgánica, Universidade de Santiago de Compostela, Jenaro de la Fuente s/n, 15782 Santiago de Compostela, Spain

**Keywords:** NMR, UV−vis, pyranoanthocyanins, dendrimers, encapsulation, pH sensors, host−guest interaction

## Abstract

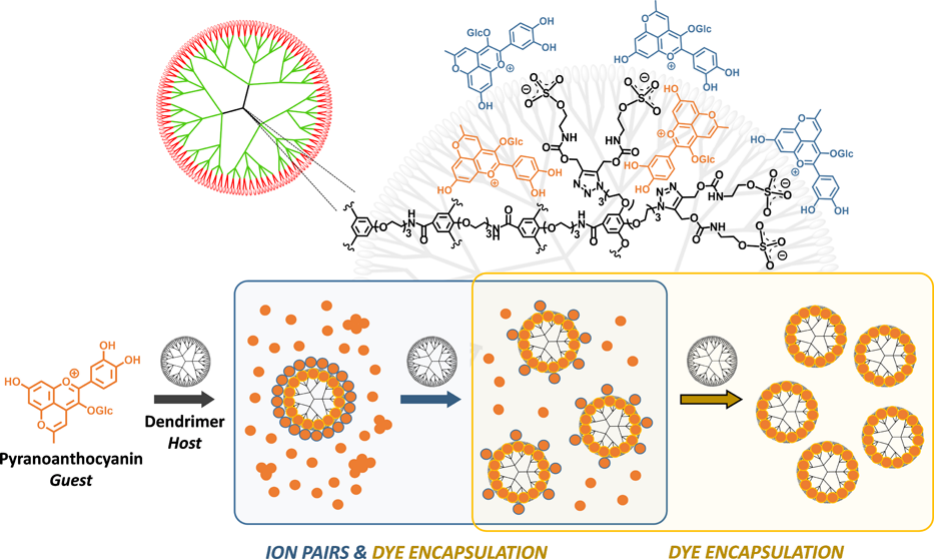

Anionic dendrimers have recently emerged as hosts (H) for the color stabilization of the flavylium cation of anthocyanin guests (G). The interaction with a promising, more hydrophobic pyranoanthocyanin illustrates how the structure and concentration of the dye modulate the host–guest interaction mechanisms. NMR and UV–vis titrations (host over guest, from G/H ratio 2089 to 45) showed that at relatively low dendrimer-to-dye concentrations, ion pairs at the dendrimer periphery prevail over dye encapsulation. This promotes the deaggregation of the dye, not previously observed with anthocyanins, and related to the more hydrophobic nature of this dye (deshielding of the dye ^1^H signals, higher *T*_2_ relaxation times, constant diffusion coefficient). As the dendrimer concentration increases, the dye encapsulation, earlier unseen with structurally simpler flavylium dyes, becomes dominant (shielding and broadening of the dye ^1^H signals and lower *T*_2_ and diffusion coefficient). The interaction parameters of the encapsulation process (*K* ∼ 4.51 × 10^4^ M^–1^, *n* ∼ 150) indicate the binding of ca. one pyranoanthocyanin molecule by each sulfate terminal group. Our results provide insights into the ability of dendrimers to host structurally diverse pyranoflavylium-based dyes and how the structure of the latter modulates the range of interactions involved. The encapsulation ability of this dendrimer to such pH-sensitive dyes is envisioned for the host–guest sensing applications such as pH-responsive systems used for example in food smart packaging.

## Introduction

**Figure 1:**
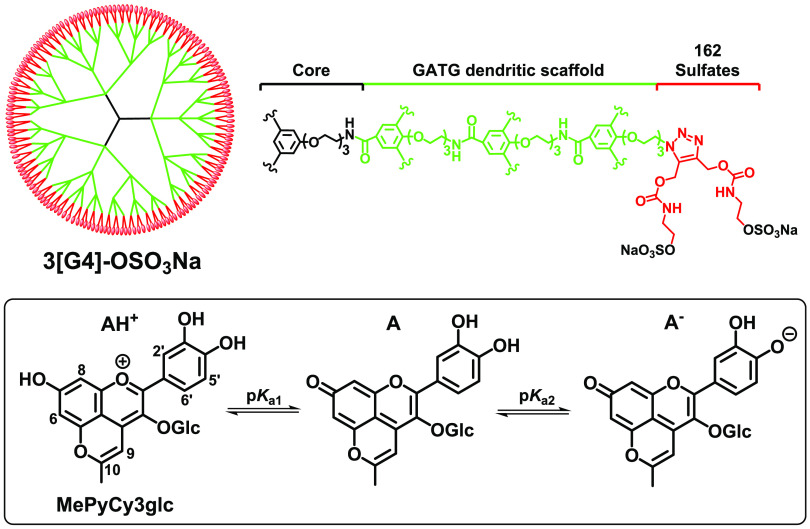
Structures of 3[G4]-OSO_3_Na dendrimer carrying 162 sulfate groups (host or H) and 10-methylpyranocyanidin-3-glucoside cation (MePyCy3glc, guest or G) and its chemical equilibrium species as a function of pH.

Flavylium-based dyes represent a huge family of natural and synthetic pigments, which include anthocyanins, 3-deoxyanthocyanins, and anthocyanin-derived pigments (e.g., pyranoanthocyanins) with interesting applications as colorants for textiles, foods, cosmetics, and smart and functional materials because of their pH and light responsiveness.^1^ Among them, pyranoanthocyanins (Figure 1) display characteristic chromatic features and a more intense orange color because of a hypsochromic shift of their λ_max_ (478–510 nm) compared to anthocyanins (516–541 nm).^2^ Their pyranic ring substitution impedes the production of hemiketal colorless species and leads to more evident color changes than anthocyanins in response to pH variations, an advantage of sensing applications.^3,4^ Furthermore, bearing their natural occurrence and high biocompatibility, these pigments find great potential as pH sensors for real-time monitoring of food spoilage. Thus, as pH increases, the flavylium cation (AH^+^) undergoes very fast proton transfer reactions, leading to the formation of neutral and anionic quinoidal bases (A and A^–^) with different colors.^4,5^

Supramolecular host–guest systems have found application from materials to the biomedical arena with the ultimate goal of improving the physicochemical properties of the guests.^6,7^ In nature, anthocyanins participate in complex supramolecular structures via different mechanisms (self-association, copigmentation, metal complexation), rendering some of the beautiful colors found in flowers.^8^ Because of their globular architecture at the nanoscale, high functional surface, and inherent multivalency, dendrimers are considered archetypal macromolecular hosts.^9−11^ The first dendrimer-based anthocyanin host has recently been described: a water-soluble, polyanionic gallic acid triethylene glycol (GATG)^12,13^ dendrimer (functionalized with 162 terminal sulfate groups) that shows a great impact on the color-stabilizing mechanisms of the red flavylium cation of anthocyanins via reversible contact ion pairs at the dendrimer periphery.^14^ In this work, a deep mechanistic study of the interaction of GATG-based dendrimers with a more hydrophobic pyranoanthocyanin-type pigment (MePyCy3glc) with promising pH-sensing applications was performed using NMR (^1^H titration, ^1^H *T*_2_ relaxation, DOSY, NOESY) and UV–vis spectroscopy (Figure 1).

## Materials and Methods

### Reagents

Theorell and Stenhagen universal buffer^15^ was prepared as described in the previous work.^14^ Cyanidin-3-glucoside chloride salt was obtained by extraction from blackberries (*Rubus fruticosus* L.). The purification process was performed as described elsewhere,^16^ and the purity of the pigment was assessed by HPLC–DAD and ^1^H NMR spectroscopy. The dendrimers were obtained by synthesis as described below. Other reagents were obtained from Sigma-Aldrich (Madrid, Spain).

### Synthesis of Dendrimers

3[G4]-OSO_3_Na with 162 terminal sodium sulfate groups (MW: 61 672 g mol^–1^) was obtained from 3[G3]-N_3_ and ammonium 4,11-dioxo-5,10-dioxa-3,12-diazatetradec-7-yne-1,14-diyl bis(sulfate) via azide-alkyne cycloaddition (91%):^14^^1^H NMR (500 MHz, D_2_O) δ: 7.26–7.12 (m, 78H), 6.26 (br s, 3H), 5.43–5.16 (m, 324H), 4.71–4.58 (m, 162H), 4.29–4.01 (m, 564H), 4.00–3.51 (m, 1038H), 3.48–3.32 (m, 324H) (Figure S8). ^13^C NMR (125 MHz, D_2_O) δ: 168.8, 157.5, 157.0, 151.8, 141.9, 139.6, 132.6, 129.3, 106.1, 72.1, 69.9, 69.5, 69.0, 68.8, 68.3, 67.2, 67.1, 57.1, 53.9, 48.6, 40.0 (Figure S9). IR (KBr): 3446, 2935, 1718, 1542, 1255 cm^–1^.

Using similar reaction conditions as above from 3[G3]-N_3_ and 10,17-dioxo-3,6,11,16,21,24-hexaoxa-9,18-diazahexacos-13-yne-1,26-diaminium chloride or but-2-yne-1,4-diyl bis((2-(2-(2-hydroxyethoxy)ethoxy)ethyl)carbamate), cationic 3[G4]-NH_2_·HCl (162 ammonium groups) and neutral 3[G4]-OH (162 hydroxyl groups) dendrimers were prepared in 92% yield as control hosts for the interaction with MePyCy3glc.

3[G4]-NH_2_·HCl: ^1^H NMR (500 MHz, D_2_O) δ: 7.16 (br s, 78H), 5.40–5.12 (m, 324H), 4.71–4.55 (m, 162H), 4.29–4.02 (m, 240H), 4.00–3.46 (m, 2334H), 3.34–3.17 (m, 648H) (Figure S10). ^13^C NMR (125 MHz, D_2_O) δ: 168.7, 157.6, 157.0, 151.9, 141.9, 139.7, 132.7, 129.2, 106.2, 72.0, 69.8, 69.7, 69.4, 69.0, 68.7, 68.3, 66.2, 56.9, 53.7, 48.5, 39.8, 38.9 (Figure S11). IR (KBr): 3383, 2971, 1677, 1201, 1130 cm^–1^.

3[G4]-OH: ^1^H NMR (500 MHz, D_2_O) δ: 7.24–7.10 (m, 78H), 5.40–5.13 (m, 324H), 4.74–4.58 (m, 162H), 4.26–4.00 (m, 240H), 4.00–3.40 (m, 2658H), 3.34–3.18 (m, 324H) (Figure S12). ^13^C NMR (125 MHz, D_2_O) δ: 168.4, 157.6, 157.2, 152.1, 141.9, 139.8, 132.7, 129.2, 106.1, 72.1, 71.6, 69.8, 69.4, 69.2, 69.0, 68.8, 68.5, 68.3, 60.2, 56.8, 53.6, 48.5, 39.9 (Figure S13). IR (KBr): 3332, 2876, 1708, 1246, 1100 cm^–1^.

### Synthesis of 10-Methylpyranocyanidin-3-glucoside Chloride Salt (MePyCy3glc)

Cyanidin-3-glucoside chloride salt (50 mg, 0.10 mmol) was dissolved in a solution of acetone/water 20:80 (v/v). The reaction mixture was stirred at 37 °C and pH 2.9 for 8 days. The crude product was first prepurified in a Buchner funnel loaded with RP-18 silica gel and the fraction containing the product was eluted with MeOH/water 20:80 (v/v) solution acidified with 0.1 M HCl. Then, the final purification was performed in a column chromatography loaded with RP-18 silica gel and the product was isolated with MeOH/water 15:85 (v/v) solution acidified with 0.1 M HCl. After solvent evaporation, the product was freeze-dried and a dark orange powder was obtained with 46 % of yield.

^1^H NMR (600.13 MHz, DMSO-*d*_6_/TFA 9:1), δ (ppm): 7.93 (dd, 2.3/8.6 Hz, 1H, H-6′B), 7.80 (d, 2.3 Hz, 1H, H-2′B), 7.27 (s, 1H, H9), 7.18 (d, 1.9 Hz, 1H, H-8A), 7.08 (d, 1.9 Hz, 1H, H-6A), 6.91 (d, 8.6 Hz, 1H, H-5′B), 2.58 (s, 3H, CH_3_); *Glucose*, 4.61 (d, 7.8 Hz, 1H, H-1″), 3.51 (*, 1H, H-6a″), 3.45 (t, 8.1 Hz, 1H, H-2″), 3.28 (dd, 6.0/11.5 Hz, 1H, H-6b″), 3.18 (t, 8.8 Hz, 1H, H-3″), 3.13 (t, 9.0 Hz, 1H, H-4″), 3.03 (m, 1H, H-5″); ^13^C NMR (125.77 MHz, DMSO-*d*_6_/TFA 9:1), δ (ppm): 171.9 (C10), 167.0 (C7), 162.1 (C2), 153.5 (C5), 152.3 (C8a), 152.3 (C4′), 145.5 (C3′), 132.8 (C3), 124.9 (C6′), 120.1 (C1′), 117.1 (C2′), 116.2 (C5′), 107.5 (C4a), 102.0 (C9), 100.3 (C8), 100.1 (C6), 21.5 (CH_3_), C4 not assigned; *Glucose*, 104.3 (C-1″), 77.9 (C-5″), 76.5 (C-3″), 74.0 (C-2″), 70.0 (C-4″), 61.5 (C-6″). LC–DAD/ESI-MS: [M]^+^*m*/*z* 487, λ_max_ 472 nm. ^1^H NMR spectrum, 2D COSY, 2D HSQC, and 2D HMBC spectra of MePyCy3glc are presented in Figures S4–S7, respectively.

### HPLC–DAD/LC–DAD/ESI-MS

The analyses were performed according to the procedures described elsewhere.^17^

### Dynamic Light Scattering (DLS)

DLS measurements were performed on a Malvern Nano ZS (Malvern Instruments, U.K.), operating at 633 nm with a 173° scattering angle at 25 °C. DLS mean diameters were obtained from the volume particle size distribution provided by Malvern Zetasizer Software. DLS size is reported from the intensity particle size distributions.

### NMR Spectroscopy

Unless otherwise noted, NMR experiments were recorded on a Bruker Avance III 600 HD spectrometer operating at 600.13 MHz for ^1^H and 150.92 MHz for ^13^C, equipped with 5 mm CryoProbe Prodigy and pulse gradient units, capable of producing magnetic field pulsed gradients in the *z*-direction of 50 G cm^–1^. The NMR measurements have been done with standard BRUKER pulse sequences, in deuterium oxide (D_2_O), at 300 K and at pD 1.4. ^1^H NMR experiments were performed with water suppression using excitation sculpting with gradients (Hwang and Shaka, 1995), the acquisition time of 1.36 s, relaxation delay of 2 s, and 128 or 256 transients of the spectral width of 10 000 Hz collected into 32 K time domain points.

#### NOESY

Typical measuring conditions for the ^1^H/^1^H and 2D NOESY recorded in the phase-sensitive mode and with water suppression were relaxation delay of 2 s, 64–128 scans, and a total 2 K data points in F2 and 256 data points in F1 over a spectral width of 10 000 Hz. NOESY experiments were carried out using a mixing time of 100, 200, 400, 500, 700, and 800 ms in the phase-sensitive mode.

#### DOSY

Proton-registered diffusion-ordered NMR (^1^H DOSY) experiments were performed using the bipolar longitudinal Eddy current delay (BPPLED, bipolar pulsed field gradient longitudinal Eddy delay) pulse sequence as described in the literature.^18^ The experimental conditions of DOSY experiments were set according to the previous work.^19^ The diffusion coefficients (*D*) were obtained by measuring the signal intensity of the methyl protons of the guest.

#### NMR Relaxation

^1^H *T*_2_ relaxation experiments were measured at 300 K in a 11.7 T Bruker DRX-500 spectrometer (^1^H frequency 500 MHz) equipped with a BBI probe with PFG gradients on the *z*-axis using the Carr–Purcell–Meiboom–Gill (CPMG)^20,21^ pulse sequence with presaturation. After an initial relaxation delay (*d*_1_) of 7 s, the presaturation was applied during 4 s at the frequency of the residual HOD peak of the solvent (∼4.7 ppm) by a continuous wave pulse of low power. A total of 16 durations (*t*) of the CPMG were explored by a repetition of the single CPMG block with an interpulse delay of 1.4 ms. The durations (*t*) were explored in independent fids from 1.4 ms up to a maximum of ca. 5 times the largest ^1^H *T*_2_. The fids were acquired as a pseudo-2D spectrum. Each fid acquisition time (aq) was 1.47 s, and the spectral width was 10.6 ppm. A total of 24 scans were acquired for each fid. The signal integral (*I*) at each value of *t* was fitted to the monoexponential eq 1 to determine the relaxation time *T*_2_
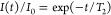
1where *I*(*t*) and *I*_0_ are the observed signal integrals at a given value of *t* and for *t* = 0, respectively. OriginPro 8.5 Software (OriginLab Corporation) was used to perform the exponential fittings to obtain the relaxation times *T*_2_.

#### ^1^H NMR Titration

For the NMR titration, a 261 μM solution of MePyCy3glc was prepared in D_2_O, with the pH was adjusted to 1 (pD 1.4), and transferred to 5 mm NMR tubes. Sodium trimethylsilyl-[2,2,3,3-d4]-propionate (TSP, 5 μL, 2 mg mL^–1^ in D_2_O) was used as an internal standard for chemical shift measurements. Successive volumes of dendrimers stock solutions in D_2_O at pD 1.4 were added to the NMR tube to obtain different guests: dendrimer molar ratios (G/H) during the titration. All ^1^H NMR spectra were recorded under the same experimental conditions of the previous work.^14^

The chemical shift variations (Δδ_obs_) of guest protons as a function of the G/H ratio can be expressed through eq 2(9)
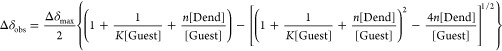
2Δδ_max_ is the maximum chemical shift variation of the guest molecule in the NMR titration experiment and *K* is the binding affinity or association constant. The number of binding sites (*n*) was obtained by fitting the titration data with eq 2 using a nonlinear least-squares method within the software program Microsoft Excel.

### UV–vis Spectroscopy

#### p*K*_a_ Determination

MePyCy3glc stock solution at 191 μM and dendrimer stock solution at 84.3 μM were prepared in water with 0.1 M HCl. In a plastic cell with 1 cm of optical path, 300 μL of the Theorell and Stenhagen universal buffer at pH 1, 300 μL of a 0.1 M NaOH solution, 150 μL of the dendrimer stock solution, and 150 μL of the dye stock solution (the dye and dendrimer final concentration were 32 and 14 μM, respectively) were added. In the case of the titration in the absence of the dendrimer, 150 μL of 0.1 M HCl solution was used. The titrations were performed until pH 12 by the addition of small volumes of NaOH 1 M solution. Successive spectra were recorded immediately after the addition of the base in a Thermo Scientific Evolution Array UV–vis spectrophotometer at 25 °C. The titrations were performed in triplicate. Final volumes were appropriately corrected. All pH measurements were made in a Radiometer Copenhagen PHM240 pH/ion meter. The fitting for p*K*_a_ determination was carried out using Solver program from Microsoft Excel.

The protonation/deprotonation reactions can be accounted to the global process through eqs 3–5
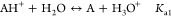
3
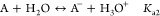
4
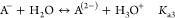
5where
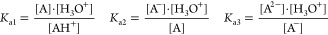
6

The total concentration of individual species, *C*_0_, is given by eq 7
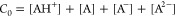
7and the mole fraction distribution of the different species, χ_*i*_, was calculated as follows
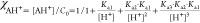
8
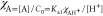
9
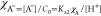
10
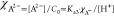
11

The p*K*_a_ values were determined by fitting the experimental data (absorbance as a function of pH) at a given wavelength using eq 12.
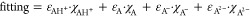
12where ε_AH^+^_, ε_A_, ε_A^–^_, and ε_A^2–^_ are the mole absorption coefficients of the individual species or components at the considered wavelength.

#### Apparent Association Constant

The apparent association constant (*nK*) of the complex MePyCy3glc–3[G4]-OSO_3_Na dendrimer was estimated by UV–vis spectroscopy in aqueous solutions at pH 1. A solution of MePyCy3glc (47.8 μM) was prepared in 0.1 M HCl (solution A). Similarly, a solution containing a mixture of MePyCy3glc (47.8 μM) and 3[G4]-OSO_3_Na dendrimer at the concentration of 6.32 μM was prepared (solution B). Then, to solution A, a known volume of solution B was added, allowing us to achieve increasing concentrations of dendrimer (from 0.06 to 1.26 μM). UV–vis absorption spectra were recorded in a Thermo Scientific Evolution Array UV–vis spectrophotometer from 360 to 830 nm in a 1 cm path length cell. The absorbance values variations (Abs) as a function of dendrimer concentration [*L*_0_] can be expressed by eq 13, previously developed for similar host–guest interactions^22^
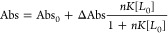
13where *nK* is the apparent binding constant, *n* is the number of nonspecific binding sites of the host where guest can bind, and [*L*_0_] the total concentration of the dendrimer added. The data could be fitted by eq 13 using the nonlinear least-squares method and the Solver function from Microsoft Excel.

## Results and Discussion

### NMR Titration Reveals Two Interaction Modes

**Figure 2:**
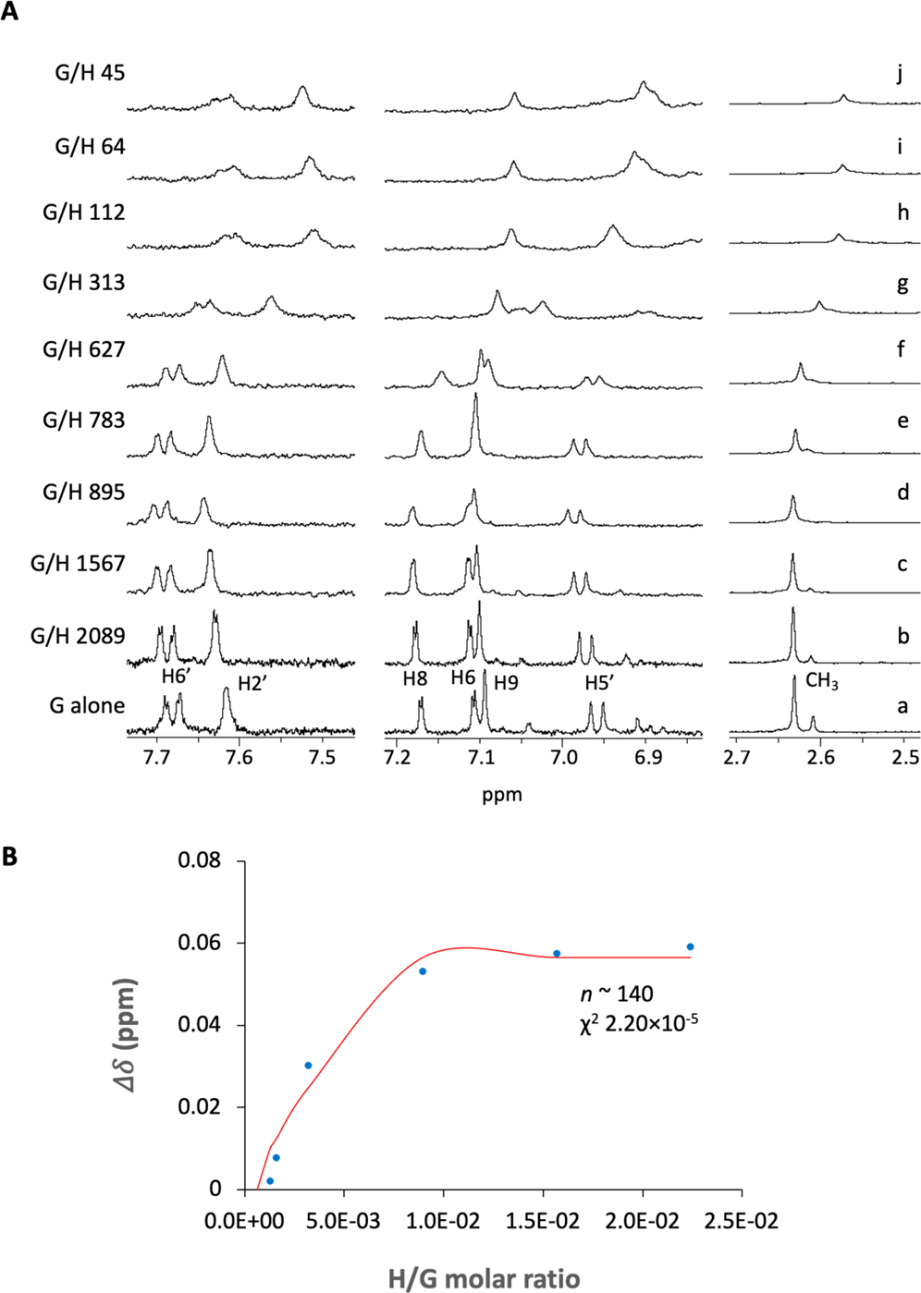
(A) ^1^H NMR titration of MePyCy3glc (261 μM) with increasing concentrations of host dendrimer: (a) no dendrimer; (b) 0.12 μM; (c) 0.16 μM; (d) 0.29 μM; (e) 0.33 μM; (f) 0.41 μM; (g) 0.80 μM; (h) 2.2 μM; (i) 3.6 μM; and (j) 5.0 μM. (B) Chemical shift variation of MePyCy3glc methyl protons as a function of H/G molar ratio.

The interaction between the dendrimer host (H) and the MePyCy3glc guest (G) was first studied by NMR spectroscopy. A ^1^H NMR titration was performed in D_2_O at pD 1.4. The chemical shift variations (Δδ_obs_) of the guest (261 μM) protons were followed with increasing concentrations of the dendrimer (Figure 2A). At very low relative concentrations of dendrimer (from 0.12 to 0.29 μM, G/H ratios 2089–895), all aromatic and methyl protons of the guest were shifted downfield (deshielding effect caused by the decrease of the electron density around the hydrogen nucleus^23^), suggesting the formation of reversible contact ion pairs between the pyranoflavylium cation and the anionic sulfate groups at the periphery of the dendrimer, an interaction resembling those of anthocyanins^14^ and other host–guest systems with dendrimers.^9,24,25^ At higher dendrimer concentrations (from 0.33 to 2.2 μM, G/H 783–112), a larger upfield shift of the guest protons was observed (shielding effect caused by the increase of the electron density around the hydrogen nucleus^23^), accompanied by a decrease in the intensity of the signals. This trend, not observed with anthocyanins, is compatible with guest molecules penetrating the internal periphery of the dendrimer,^9^ an encapsulation near the anionic surface facilitated by the hydrophobicity of the dye. Interestingly, when control NMR titrations were performed with dendrimers of a similar size and an identical number of peripheral cationic (ammonium) or neutral (hydroxyl) groups, no effect on the chemical shifts of MePyCy3glc were observed (Figures S2 and S3 in the SI), pointing to a selective interaction with the anionic dendrimer.

The number of guest molecules (*n*) able to bind to the host during the second part of the NMR titration (shielding; G/H 783–45) was estimated by fitting the data to eq 2,^9^ where Δδ_max_ is the maximum chemical shift variation of the guest molecule and *K* is the binding affinity. As shown in Figure 2B for the methyl protons, a Δδ_max_ plateau was reached after the addition of ca. 3.6 μM of the dendrimer. This afforded a number of binding sites around 140, which represents ca. 1 guest bound to each sulfate group.

### UV–vis Spectroscopy Analysis of the Dye Encapsulation

**Figure 3:**
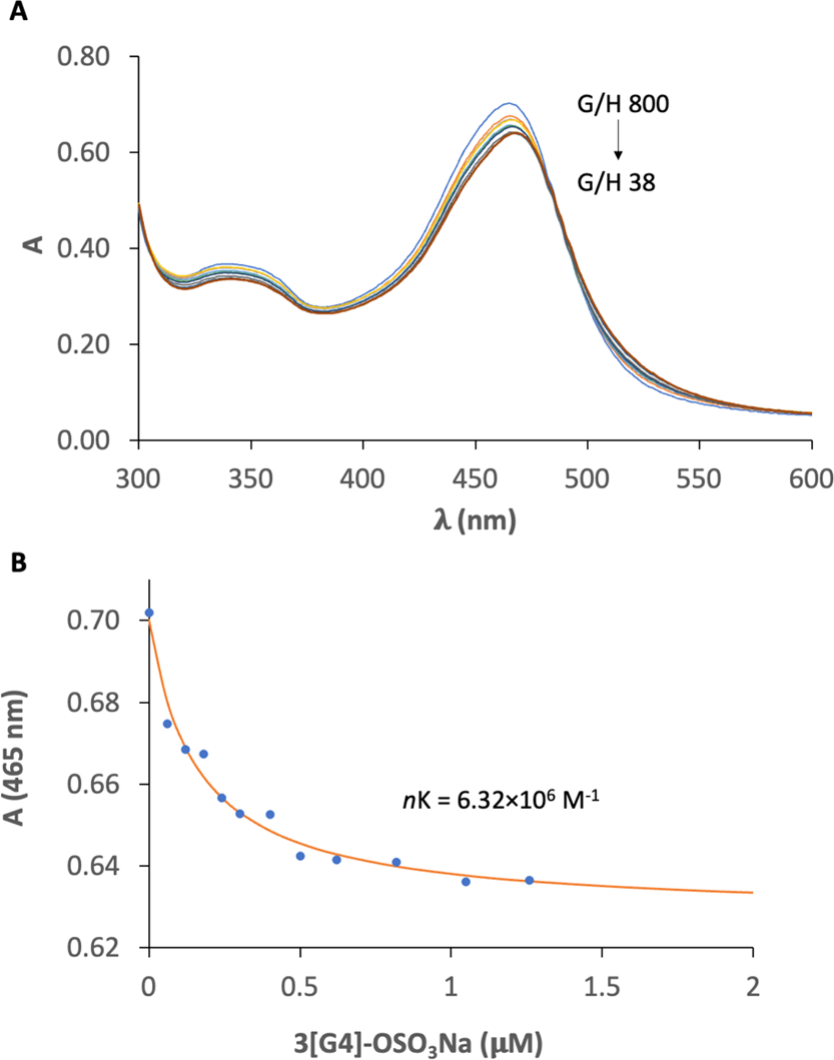
(A) UV–vis titration of MePyCy3glc guest (48 μM) with increasing concentrations of 3[G4]-OSO_3_Na dendrimer (from 0.06 to 1.26 μM) in H_2_O at pH 1. (B) Fitting the data using eq 13.

Confirmation of the encapsulation behavior was obtained from UV–vis spectroscopy titration, where the interaction of MePyCy3glc at a fixed 48 μM concentration was studied (H_2_O, pH 1) by successive additions of the dendrimer (from 0.06 to 1.26 μM, G/H 800–38). A small decrease in the absorbance intensity was revealed along with a bathochromic shift (ca. 7 nm) of its maximum wavelength (Figure 3A), effects that are compatible with the cationic guests located at the dendrimer hydrophobic internal periphery as observed for other nondendritic, flavylium host–guest systems.^26,27^ From Figure 3B, it was possible to fit the data (eq 13) and obtain an apparent binding constant *nK* of 6.32 × 10^6^ M^–1^. From this and the number of binding sites determined by NMR, the binding constant *K* for the encapsulation process has been estimated to be ca. 4.51 × 10^4^ M^–1^. This value is the in the same order of magnitude as the one obtained for the interaction between cyanidin-3-glucoside and a low methyl esterified fraction of pectic polysaccharides recently reported.^28^

### DOSY, NOESY, and *T*_2_ Relaxation

To understand the host–guest interaction phenomena along the titration, diffusion-ordered NMR spectroscopy (DOSY), nuclear overhauser effects (NOESY), and transverse relaxation (*T*_2_) experiments were performed. Indeed, NMR is a powerful tool to study intermolecular interactions, where the variations of the chemical shifts, diffusion, NOE, and relaxation properties of the guest upon macromolecular binding can be exploited as a measure of the process.^29^

**Figure 4:**
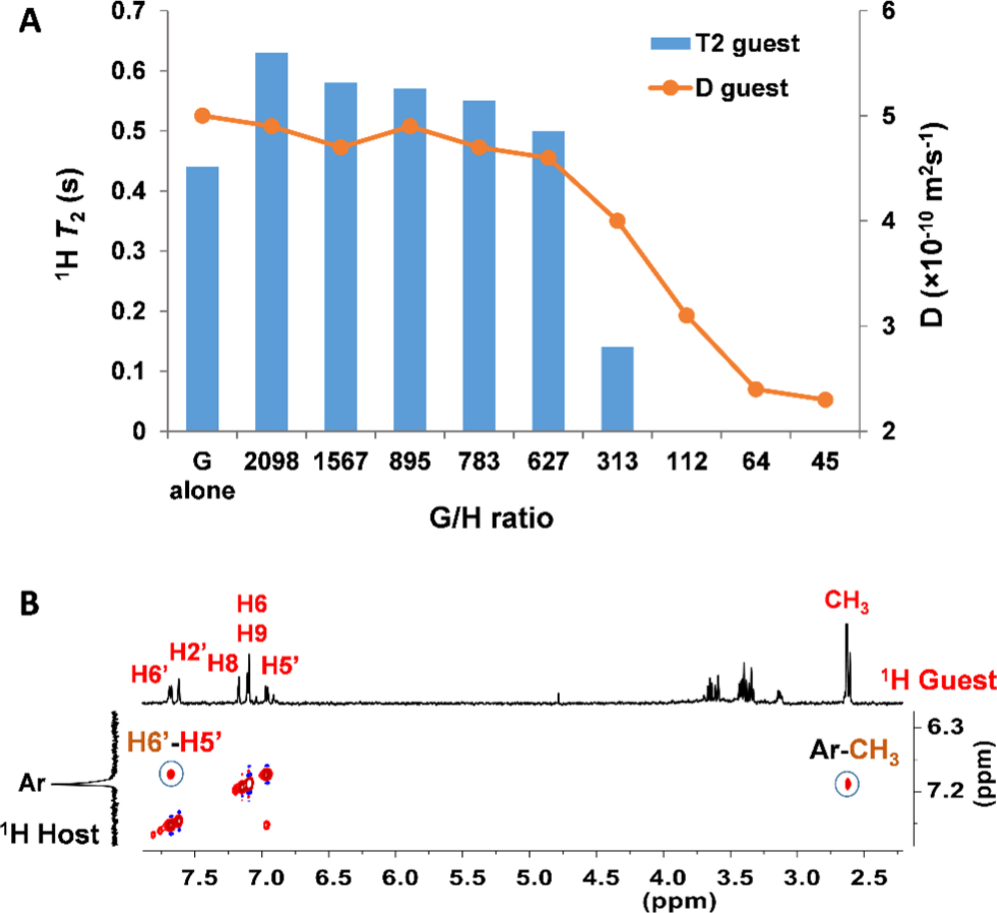
(A) Diffusion coefficients (*D*) and transverse relaxation times (^1^H *T*_2_) of MePyCy3glc (methyl protons, 261 μM) obtained after successive additions of dendrimer (G/H 2089–45). (B) NOESY spectrum showing a correlation between protons of the guest and the dendrimer (G/H 627).

Since the molecular weight of the host dendrimer is ca. 100 times higher than that of the guest, their diffusion coefficients (*D*) constitute a useful tool to assess the interaction. As expected, the dendrimer alone revealed a slower *D* = 0.56 × 10^–10^ m^2^ s^–1^ (8 μM) than the dye *D* = 5.1 × 10^–10^ m^2^ s^–1^ (261 μM). In the case of a supramolecular host–guest interaction, both species in the complex should diffuse at the slower rate of the host, effectively reducing the diffusion of the guest. The diffusion coefficient of MePyCy3glc (261 μM) was measured after successive additions of the dendrimer (G/H 2089–45). As shown in Figure 4A, the diffusion coefficient of the guest did not change during the first part of the titration (which coincided with the deshielding effect) and started to decrease continuously only after a G/H ratio of 783 was reached (marking the deshielding–shielding transition). In the first part of the titration, the dendrimer is saturated and there is still a substantial amount of pyranoflavylium cation in the aqueous phase. Below G/H values of about 313, most of the pyranoflavylium cation is bound onto the dendrimer.

To gain a deeper insight into the guest encapsulation, NOESY spectra were recorded (261 μM guest) with the increasing concentrations of dendrimer (from 0.1 to 5 μM). Again, only after the addition of 0.33 μM dendrimer (G/H 783), it was possible to see a strong correlation between the guest and dendrimer protons, namely, an intense cross-peak between the methyl of the guest and the aromatic protons of the gallic acid repeating unit (7.10 ppm) at the dendrimer scaffold (Figure 4B).

**Figure 5:**
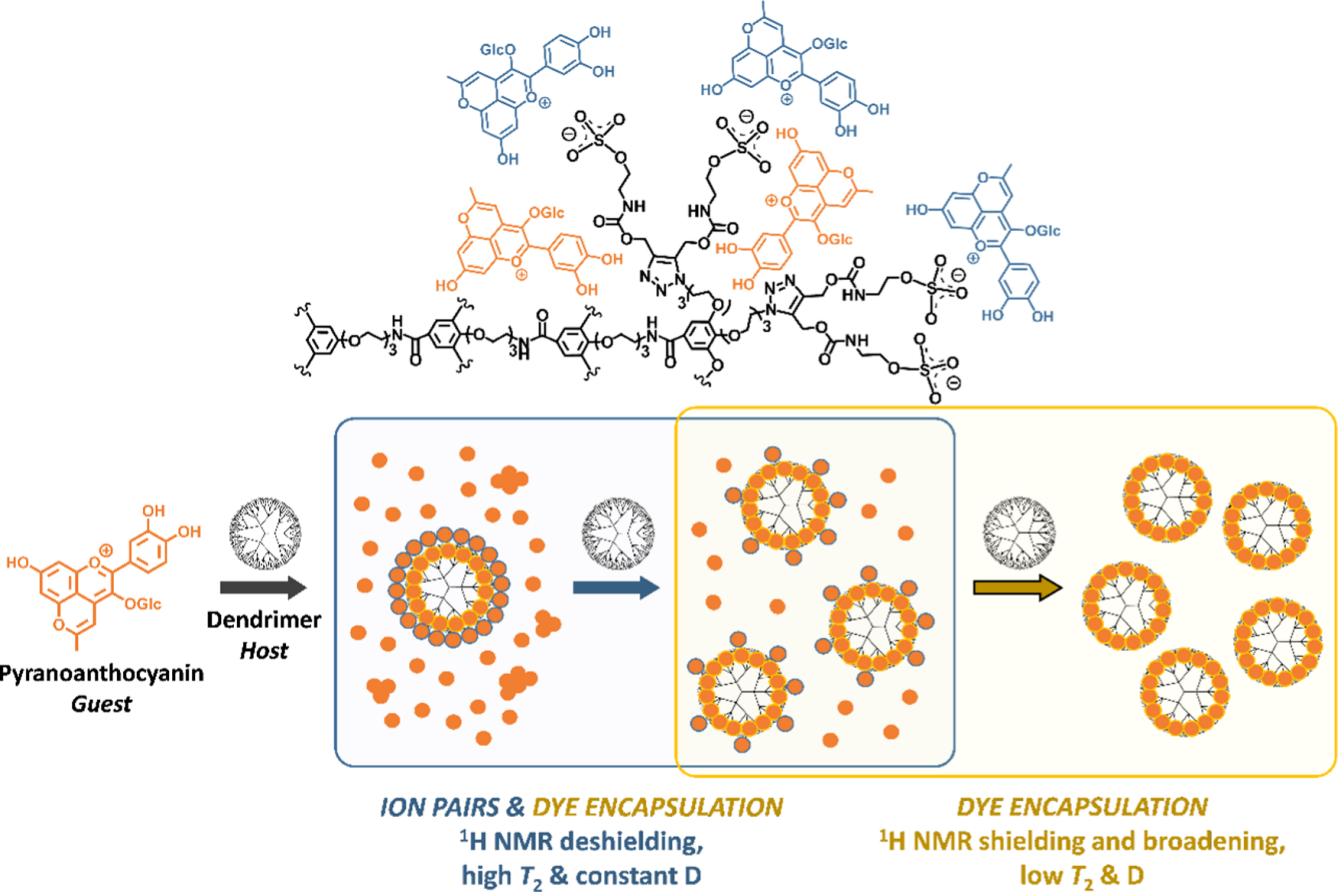
Host–guest interaction modes along the titration. At high G/H ratios, ion-paired MePyCy3glc at the dendrimer periphery prevail over dye encapsulation (and coexist with aggregated and deaggregated dye in excess). On increasing the dendrimer concentration, dye encapsulation becomes the dominant interaction.

Having confirmed the encapsulation of the MePyCy3glc into the dendrimer periphery at G/H ratios below 783 through DOSY and NOESY experiments, we performed a series of relaxation experiments to shed light on the early phases of the titration, where the guest is in large excess with respect to the host. Certainly, the reduction of transverse relaxation times (*T*_2_) of low-molecular-weight ligands binding to macromolecular receptors is often analyzed in recognition studies.^30−32^ The ^1^H *T*_2_ of the guest was analyzed by increasing the dendrimer concentration. As seen in Figure 4A for the methyl protons, the ^1^H *T*_2_ values increase after the first addition of dendrimer (G/H 2089) to level off afterward during the first part of the titration and suddenly decrease once the G/H ratios at the deshielding–shielding transition are reached (see Table S1 and Figure S1 in the SI for a whole set of ^1^H *T*_2_ data and representative magnetization decays). The initial increase and leveling of *T*_2_ values are counterintuitive. One would expect a continuous decrease of *T*_2_ associated with the reduced dynamics and overall tumbling of the guest upon binding the macromolecular host. The observed initial increase reveals an opposite behavior, which is interpreted as resulting from the deaggregation process of the dye in the presence of the dendrimer. The enhanced hydrophobic character of MePyCy3glc compared to structurally simpler anthocyanins results in a higher tendency to self-assemble in H_2_O as confirmed by dynamic light scattering analysis of a solution of the dye at the same concentration as in the NMR experiments (Figure S4). This results in lower ^1^H *T*_2_ values than that expected according to its molecular weight. As depicted in Figure 5, the addition of the anionic dendrimer triggers the deaggregation of the guest by electrostatic interactions at the dendrimer periphery as revealed by the higher ^1^H *T*_2_ values observed (characteristic of deaggregated dye) and signal deshielding (ion-paired dye). While only a small fraction of the freely solvated pyranoanthocyanin might actually internalize the host three-dimensional structure at the initial G/H ratios of the titration (not enough dendrimer available), as the dendrimer concentration increases, a larger fraction of dye will be encapsulated (lower *T*_2_ and *D*, NOESY, resonance shielding). Overall, the *T*_2_ and *D* values determined at any G/H ratio combine the contributions of aggregated, deaggregated, ion-paired, and encapsulated MePyCy3glc. This explains the observed variations of chemical shifts, *T*_2_ and *D* during the first part of the titration (Figure 5). Beyond the deshielding–shielding transition, the dendrimer concentration is so high that the encapsulation (short *T*_2_ and *D*) becomes dominant as also evidenced by the decreased intensity of the guest signals. Indeed, the inverse proportionality between ^1^H *T*_2_ and the spectral linewidth^33^ explains the broadening of the pyranoanthocyanin resonances as the titration progresses (Figure 2A).

### p*K*_a_ Determination

**Figure 6:**
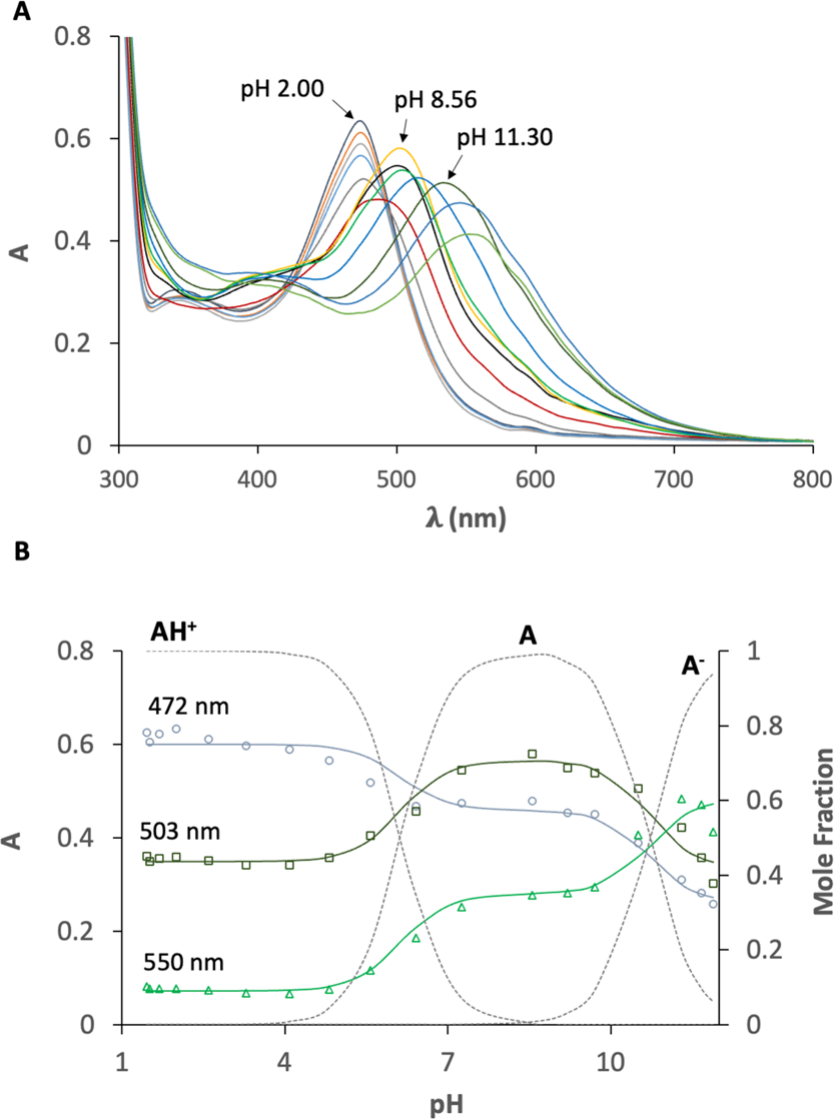
(A) UV–vis spectral variations of MePyCy3glc (32 μM) as a function of pH in the presence of dendrimer (14 μM) (pH range 2.00–11.30). (B) Fitting of the experimental data using eq 12 at a given wavelength and respective mole fraction distribution as a function of pH: p*K*_a1_ = 6.1 ± 0.1 and p*K*_a2_ = 10.7 ± 0.1.

With the aim of determining the effect of the encapsulation on the thermodynamic constants of MePyCy3glc, a UV–vis titration of a dendrimer (14 μM) and MePyCy3glc (32 μM) solution (G/H 2.3) was recorded between pH 1.5 and 12 (Figure 6A). After the fitting, two deprotonation constants were obtained: p*K*_a1_ = 6.1 ± 0.1 and p*K*_a2_ = 10.7 ± 0.1 (Figure 6B). In the absence of the dendrimer, the thermodynamic constants of the pigment were p*K*_a1_ = 5.6 ± 0.1 and p*K*_a2_ = 10.4 ± 0.1. The results indicate a color enhancement of the pigment in the presence of the dendrimer toward higher pH values and preferential stabilization of the pyranoflavylium cation species (AH^+^) rather than the neutral quinoidal base (A), increasing its first acidic constant in 0.5 pH units. The data obtained is in agreement with alternate more traditional systems, namely, anionic SDS micelles, used to tune the thermodynamic constants of similar pigments.^34^

## Conclusions

Dendrimers have recently emerged as hosts of hydrophilic anthocyanin guests to stabilize their red color. In this study, a global mechanistic perspective about the interaction with a promising, more hydrophobic pyranoanthocyanin dye (MePyCy3glc) is given, which illustrates how the structure and concentration of the dye modulate the interactions involved. NMR and UV–vis titration experiments demonstrate that at low dendrimer-to-dye concentrations, the formation of expected ion pairs at the dendrimer periphery coexists with the molecular inclusion of MePyCy3glc into the three-dimensional dendritic structure. This encapsulation, previously unseen with structurally simpler anthocyanins and promoted by the more hydrophobic nature of this dye, becomes dominant as the dendrimer-to-dye concentration increases. These results provide new insights into the ability of GATG-based dendrimers to host structurally diverse pyranoflavylium-type dyes with applications as new smart systems for real-time monitoring of pH changes of perishable foods and, therefore, to detect spoilage at early stages rather than conventional pre-estimated expiry dates.

## Supporting Information Available

Relaxation times of MePyCy3glc with the addition of dendrimer (Table S1); magnetization decay in CPMG experiments of MePyCy3glc with the addition of dendrimer (Figure S1); titrations of MePyCy3glc with control cationic 3[G4]-NH_2_·HCl host dendrimer and control neutral 3[G4]-OH dendrimer (Figures S2 and S3); ^1^H NMR, ^1^H–^1^H 2D COSY, ^1^H–^13^C 2D HSQC, and ^1^H–^13^C 2D HMBC spectra of MePyCy3glc (Figures S4–S7); and ^1^H and ^13^C NMR spectra of 3[G4]-OSO_3_Na, 3[G4]-NH_2_·HCl, and 3[G4]-OH (Figures S8–S13) (PDF)
