# Supplementary material for: Dendrimers as Color-Stabilizers of Pyranoanthocyanins: The Dye Concentration Governs the Host–Guest Interaction Mechanisms
Source: ACS Appl Polym Mater. 2021 Mar 3;3(3):1457–64. doi: 10.1021/acsapm.0c01321 (PMC8496130; doi:10.1021/acsapm.0c01321)
Supplement: Supplementary file 1 — ap0c01321_si_001.pdf [file ap0c01321_si_001.pdf]

Supporting Information

Dendrimers as Color-Stabilizers of Pyranoanthocyanins: The Dye Concentration Governs the Host-Guest Interaction Mechanisms

Luís Cruz,*[a] Juan Correa,[b] Nuno Mateus,[a] Victor de Freitas,[a] Maun H. Tawara,[b] Eduardo Fernandez-Megia*[b]

[a]REQUIMTE/LAQV, Departamento de Química e Bioquímica, Faculdade de Ciências, Universidade do Porto, Rua do Campo Alegre, s/n, 4169-007 Porto, Portugal.

[b]Centro Singular de Investigación en Química Biolóxica e Materiais Moleculares (CIQUS) and Departamento de Química Orgánica, Universidade de Santiago de Compostela, Jenaro de la Fuente s/n, 15782 Santiago de Compostela, Spain.

Corresponding authors: luis.cruz@fc.up.pt (Luis Cruz) and ef.megia@usc.es (Eduardo Fernandez-Megia)

Table S1. ^{1}H T_2 relaxation times of MePyCy3glc (261 μM) determined after successive additions of dendrimer.

G/H	7.72-7.67 (ppm) H6'	7.66-7.62 (ppm) H2'	7.19-7.16 (ppm) H8	7.13-7.08 (ppm) H6,9	6.99-6.98 (ppm) H5'	2.66-2.57 (ppm) Me
**G alone**	0.45	0.50	2.24	1.25	0.47	0.44
**2089**	0.69	0.61	2.61	1.91	0.62	0.63
**1567**	0.59	0.65	2.73	2.89	0.61	0.58
**895**	0.60	0.60	2.74	1.99	0.54	0.57
**783**	0.59	0.58	2.64	1.87	0.59	0.55
**627**	0.58	0.67	2.55	1.91	0.54	0.50
**313**	0.17	0.11	0.19	0.14	0.16	0.14

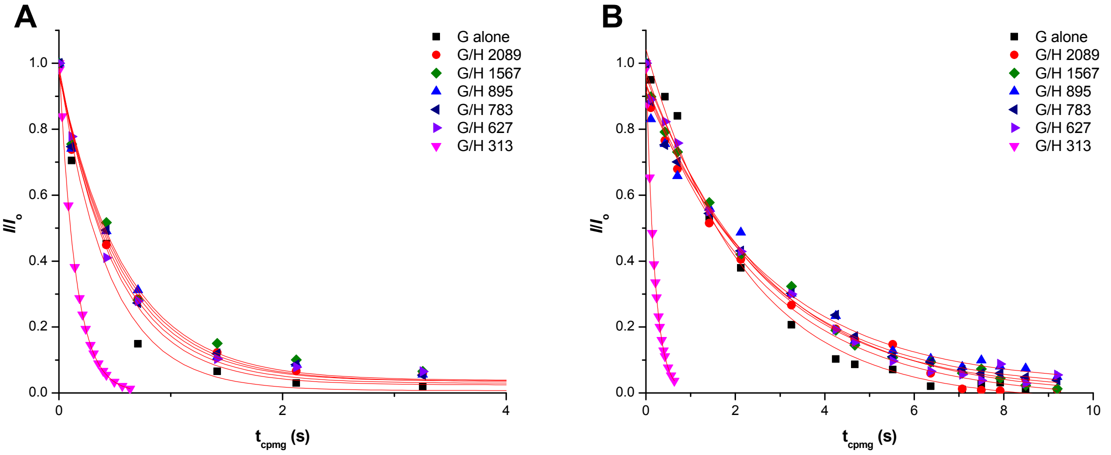

Figure S1. ^{1}H magnetization decay in CPMG experiments of MePyCy3glc (261 μM) determined after successive additions of dendrimer: (A) methyl protons, (B) H8.

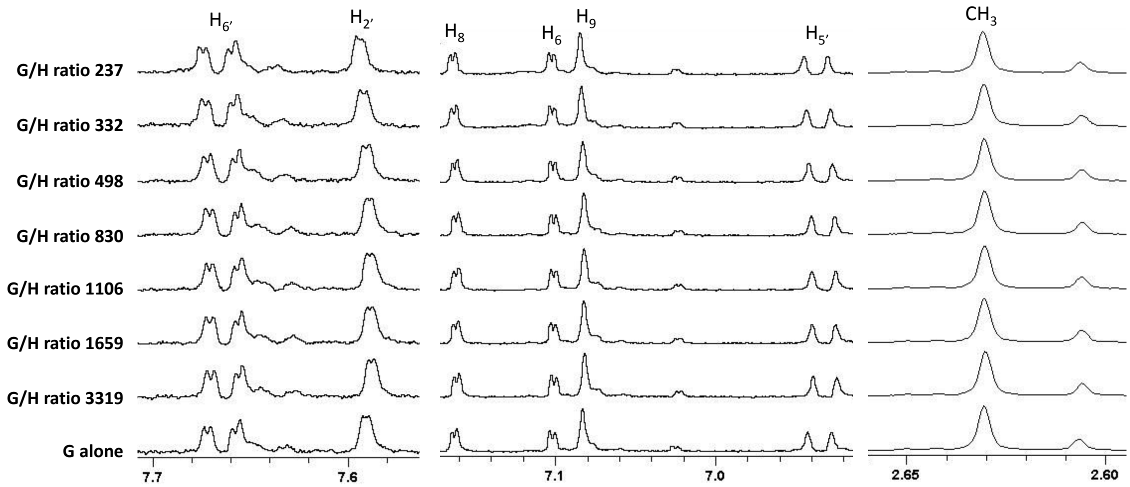

Figure S2. ^{1}H NMR titration of MePyCy3glc (261 μM) with control cationic 3[G4]-NH_2·HCl (162 ammonium groups) host dendrimer.

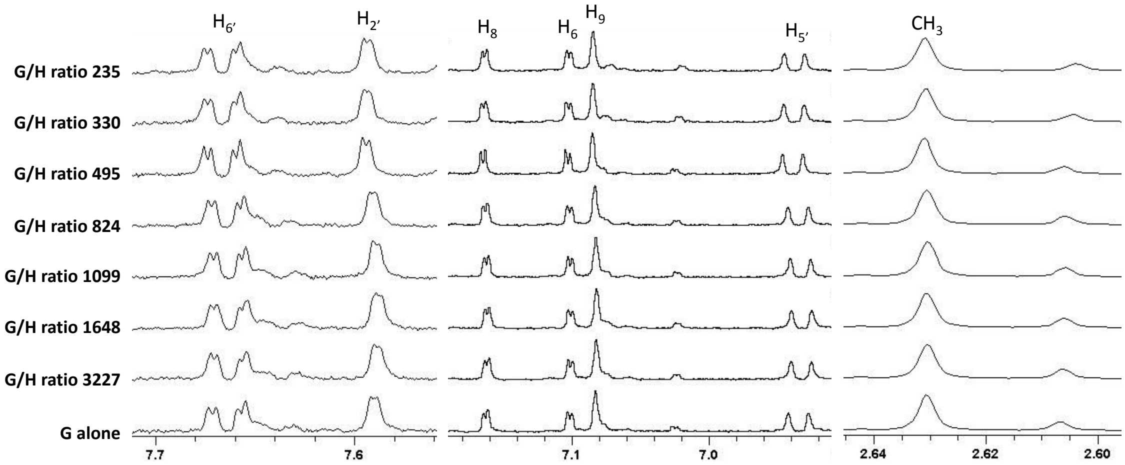

Figure S3. ^{1}H NMR titration of MePyCy3glc (261 μM) with control neutral 3[G4]-OH (162 hydroxyl groups) host dendrimer.

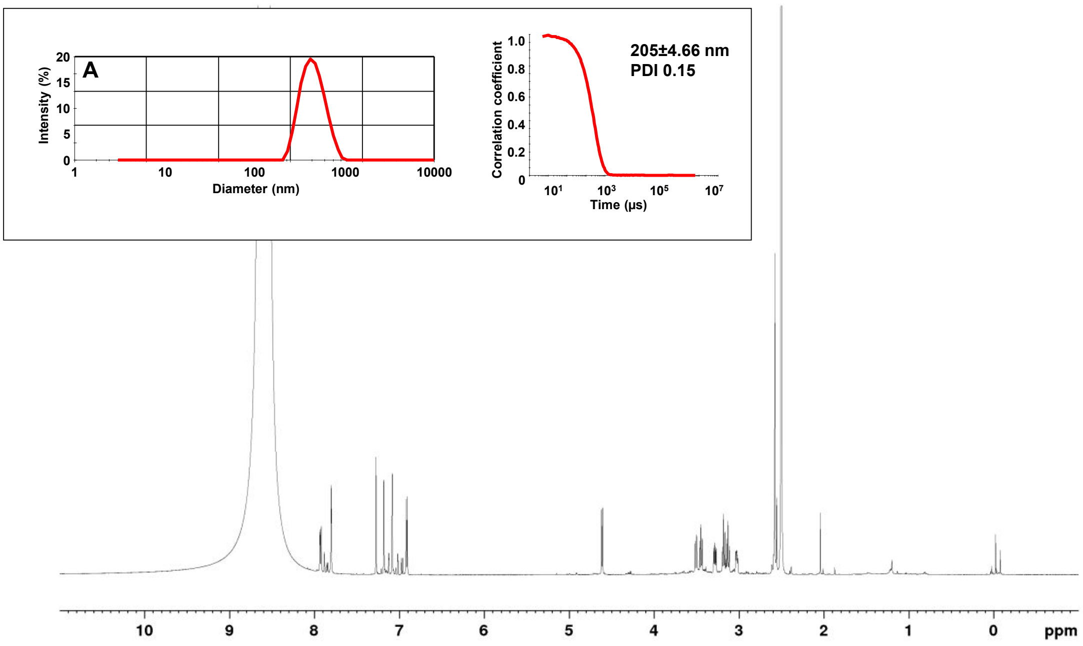

Figure S4. ^{1}H NMR (600 MHz, DMSO-d_6:TFA 9:1) spectrum of MePyCy3glc. In the inset: DLS size distribution (left) and correlation function (right) of MePyCy3glc (261 µM, 25 °C).

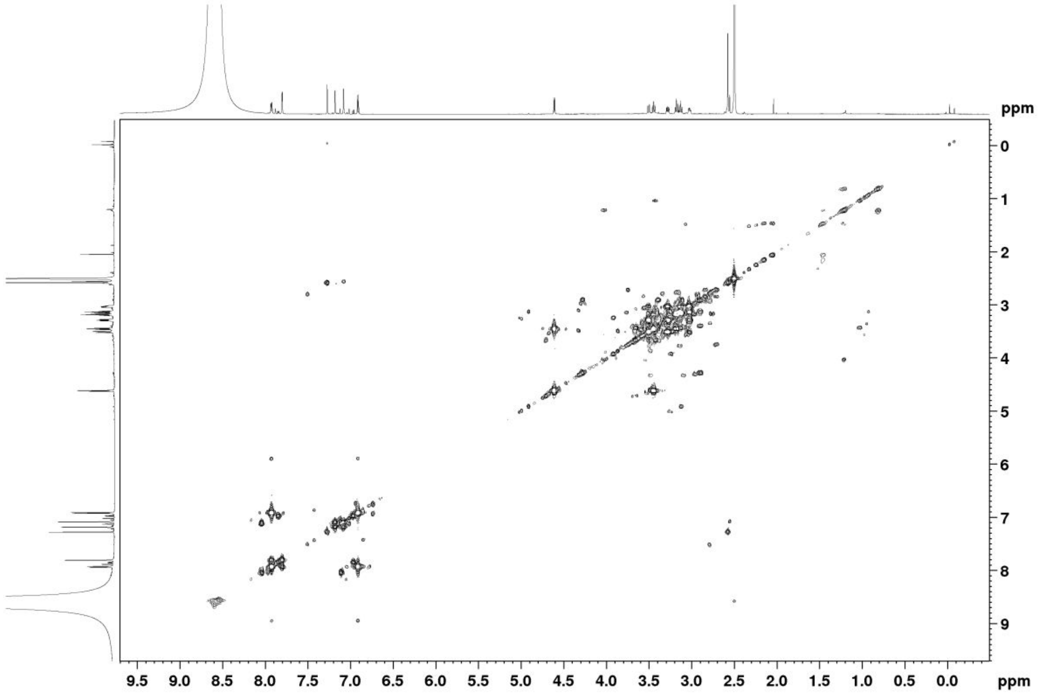

Figure S5. ^{1}H-^{1}H 2D COSY (600:600 MHz, DMSO-d_6:TFA 9:1) spectrum of MePyCy3glc.

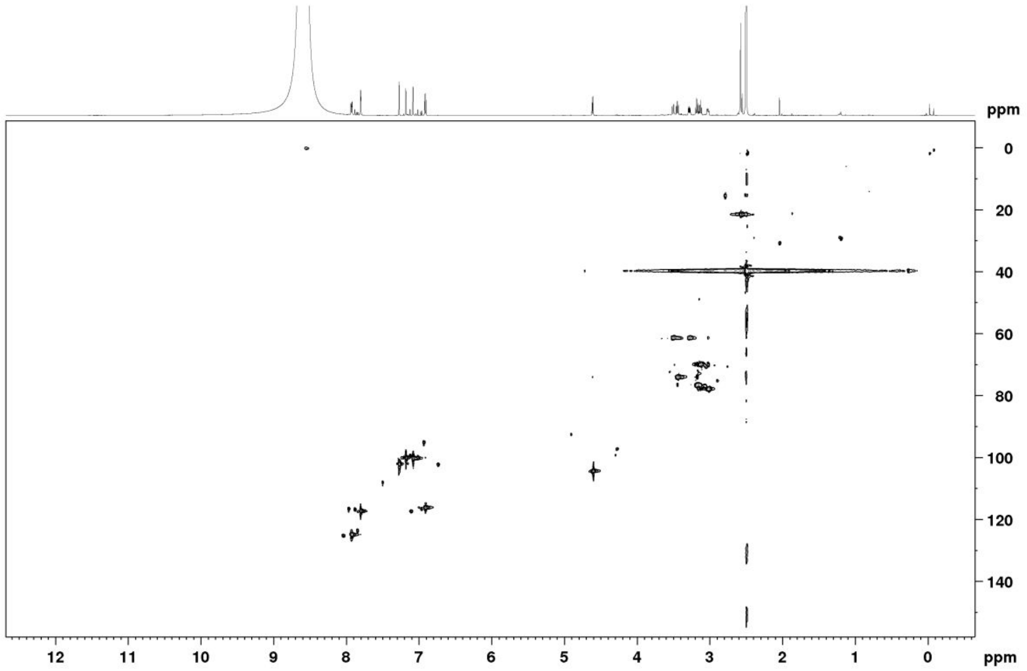

Figure S6. ^{1}H-^{13}C 2D HSQC (600:150 MHz, DMSO-d_6:TFA 9:1) spectrum of MePyCy3glc.

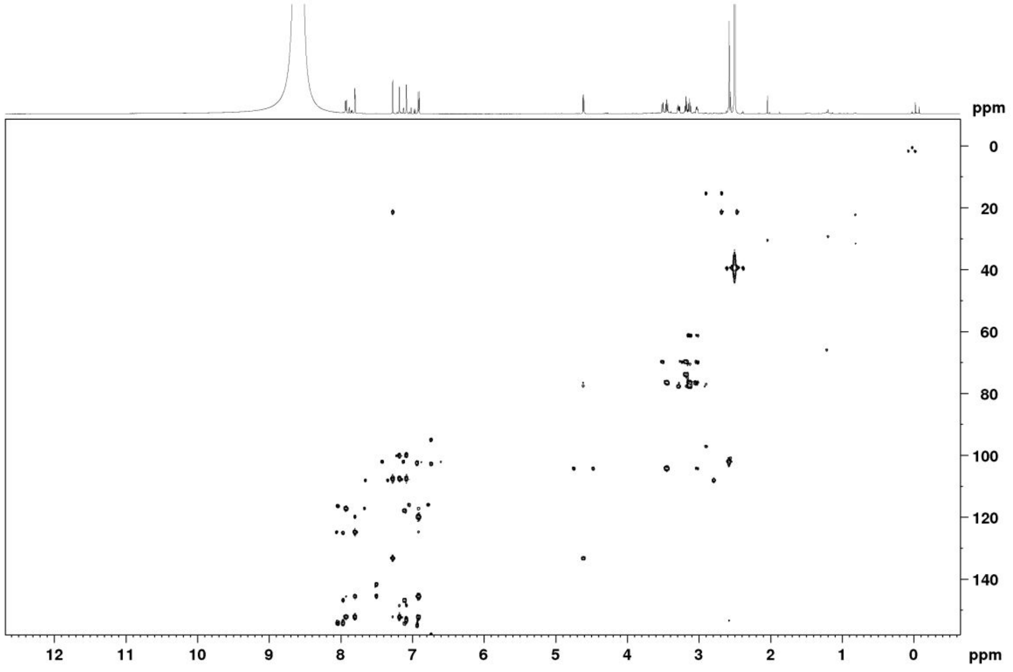

Figure S7. ^{1}H-^{13}C 2D HMBC (600:150 MHz, DMSO-d_6:TFA 9:1) spectrum of MePyCy3glc.

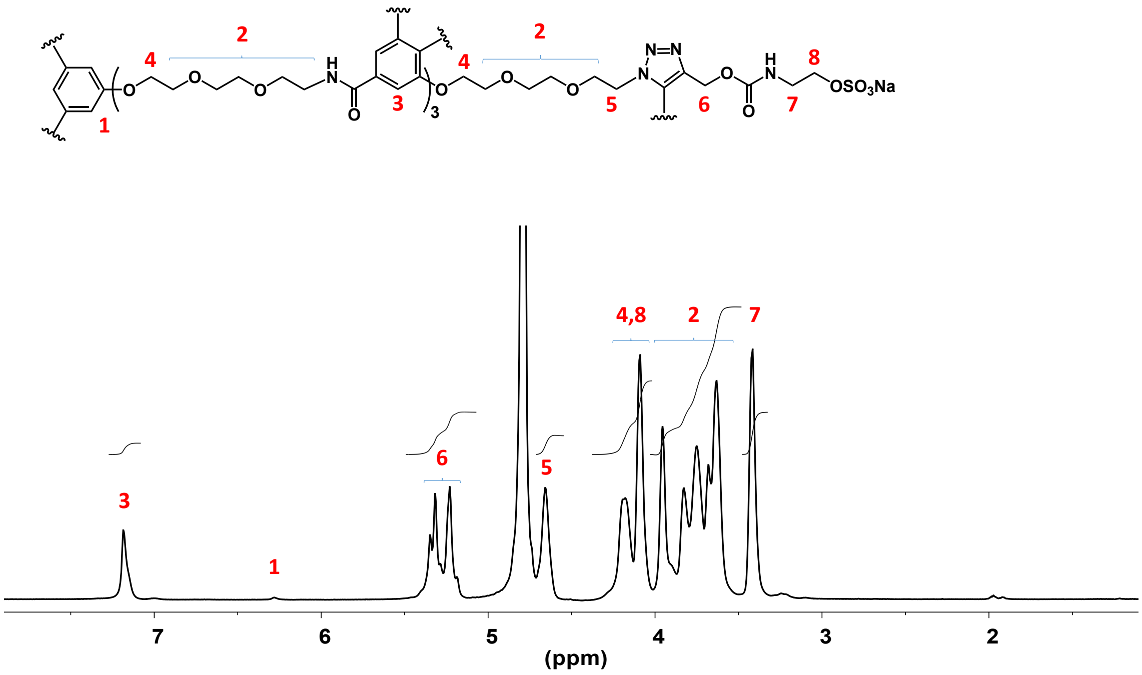

Figure S8. ^{1}H NMR (500 MHz, D_2O) spectrum of 3[G4]-OSO_3Na dendrimer.

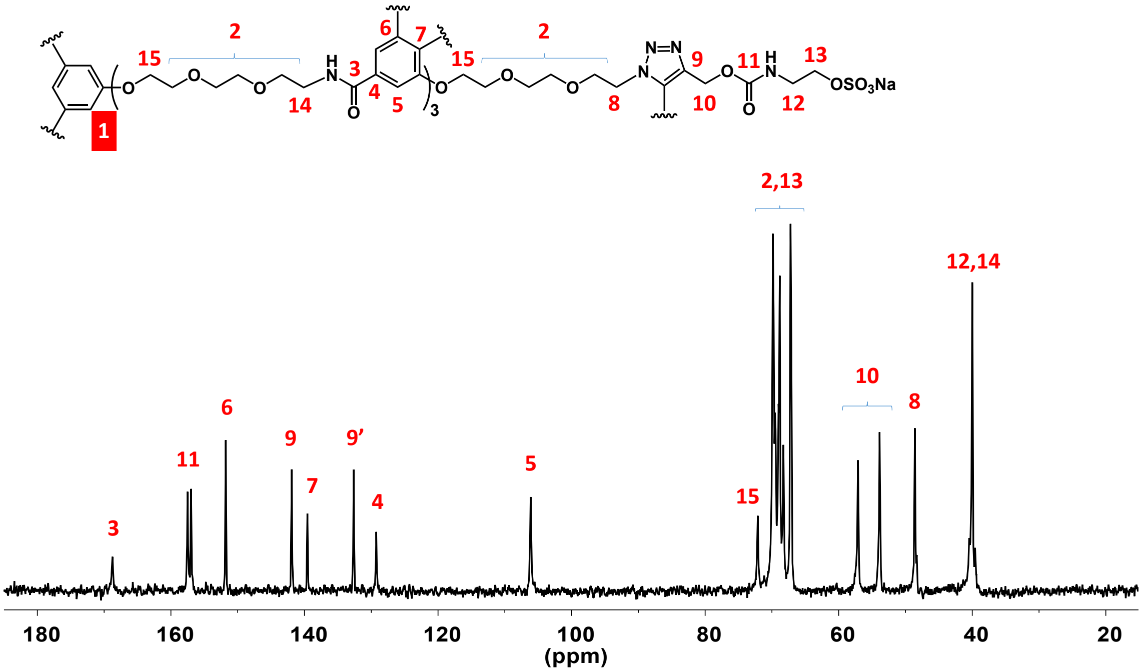

Figure S9. ^{13}C NMR (125 MHz, D_2O) spectrum of 3[G4]-OSO_3Na dendrimer.

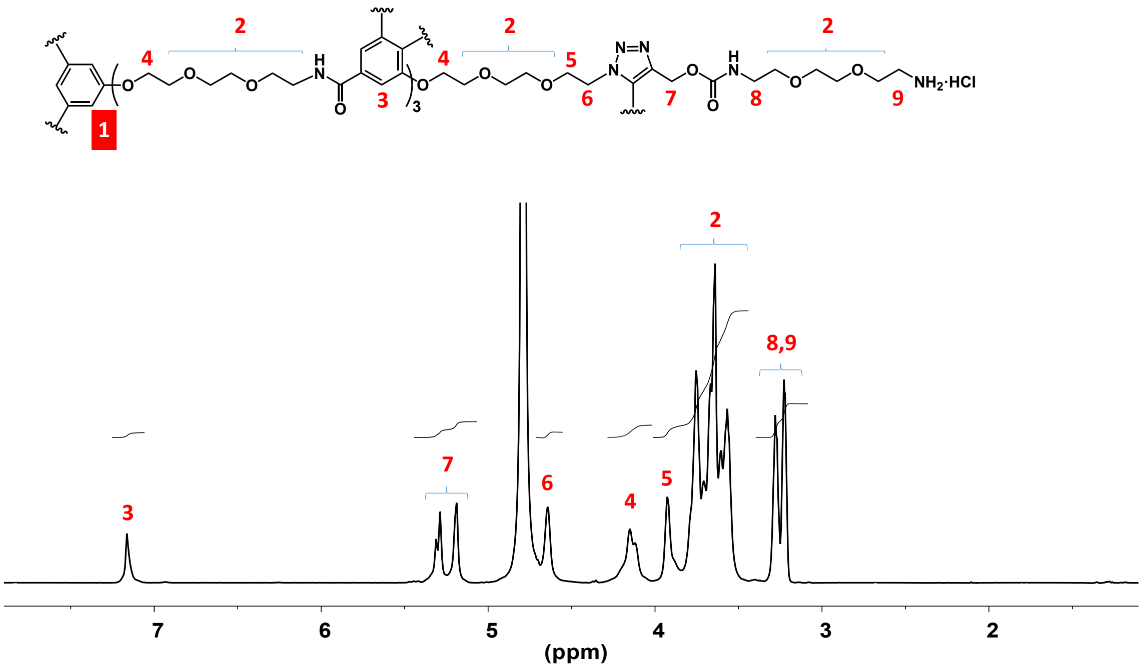

Figure S10. ^{1}H NMR (500 MHz, D_2O) spectrum of 3[G4]-NH_2·HCl dendrimer.

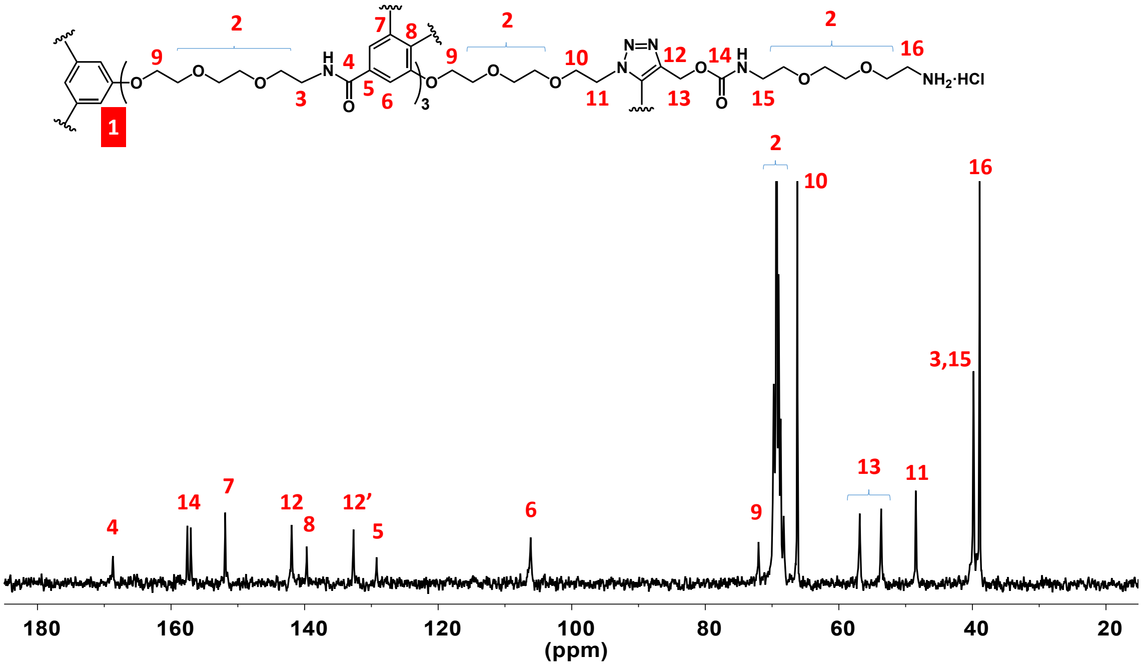

Figure S11. ^{13}C NMR (125 MHz, D_2O) spectrum of 3[G4]-NH_2·HCl dendrimer.

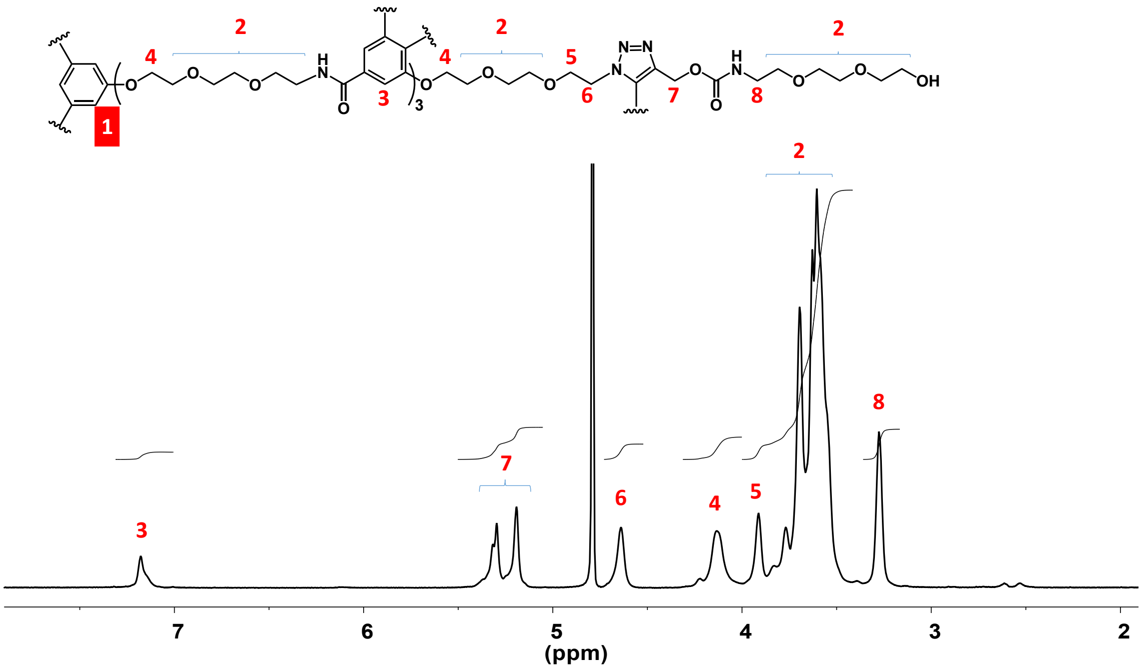

Figure S12. ^{1}H NMR (500 MHz, D_2O) spectrum of 3[G4]-OH dendrimer.

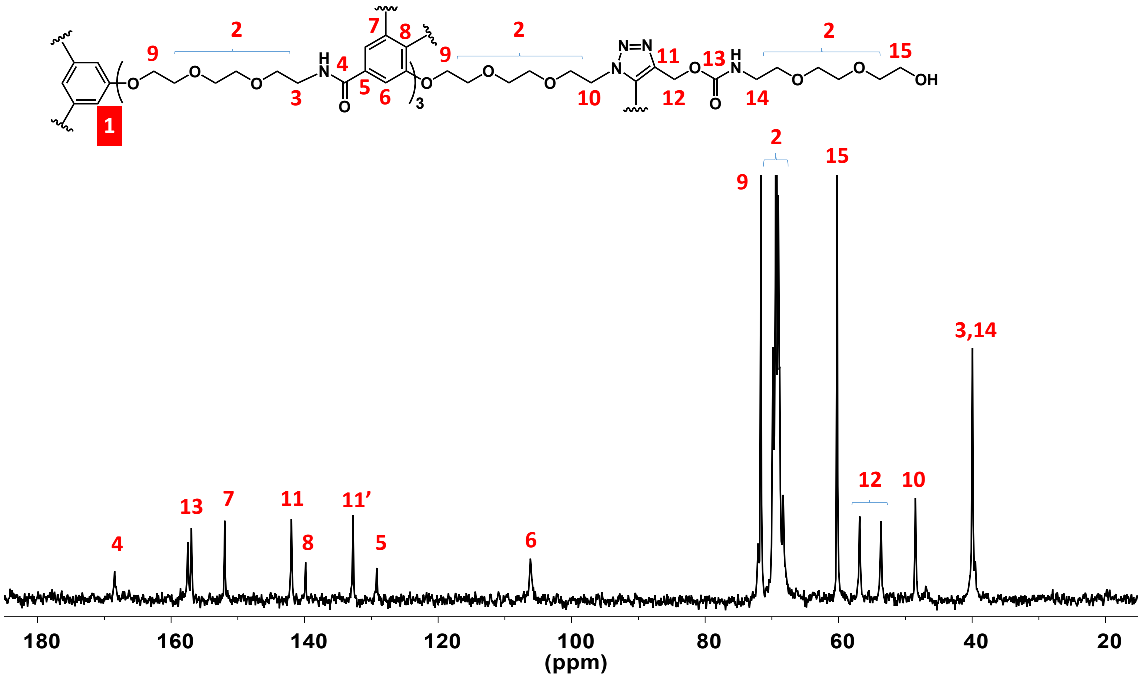

Figure S13. ^{13}C NMR (125 MHz, D_2O) spectrum of 3[G4]-OH dendrimer.
